# Protein Aggregates as Drivers of Receptor Clustering and Pathological Signaling: A Framework Linking Extracellular Amyloid‑β to Tau Dysregulation

**DOI:** 10.1021/acschemneuro.6c00331

**Published:** 2026-08-20

**Authors:** Renan C. Ratis, Juliana S. Bonini, Angelica B. W. Bold

**Affiliations:** † Postgraduation Program in Pharmaceutical Sciences, Universidade Estadual Do Centro Oeste - UNICENTRO, Guarapuava, Paraná 85040-167, Brazil; ‡ Department of Pharmacy, Universidade Estadual Do Centro Oeste - UNICENTRO, Guarapuava, Paraná 85040-167, Brazil; § Postgraduation Program in Genetics, Universidade Federal Do Paraná - UFPR, Curitiba, Paraná 80060-000, Brazil; ∥ Postgraduation Program in Internal Medicine, Universidade Federal Do Paraná - UFPR, Curitiba, Paraná 80060-000, Brazil

**Keywords:** proteinopathy, positive feedback, lipid raft, beta-amyloid, neurons

## Abstract

Extracellular protein aggregates have long been viewed primarily as toxic deposits, yet this framing fails to explain a critical clinical observation: removal of amyloid-β oligomers does not halt or reverse Alzheimer’s disease progression. Here, we propose that this paradox reflects the emergence of a self-sustaining intracellular signaling state, initiated by aggregate-driven receptor clustering but capable of persisting independently of the original extracellular trigger. We argue that oligomeric assemblies act as multivalent scaffolds that cross-link cell-surface receptors, inducing nanoscale clustering and the formation of signaling-competent membrane platforms. In Alzheimer’s disease, this mechanism links amyloid-β to a receptor complex involving PrP^c^ and mGluR5, leading to Fyn activation and downstream tau hyperphosphorylation. Critically, redistributed tau enhances Fyn recruitment to postsynaptic compartments, establishing a positive feedback loop in which kinase activity and tau pathology become mutually reinforcing, and progressively decoupled from the initiating amyloid signal. This self-amplifying circuit provides a mechanistic basis for the limited efficacy of amyloid-targeted therapies and reframes the therapeutic window problem in neurodegeneration. Beyond Alzheimer’s disease, we propose that clustering-driven feedback loops may represent a generalizable principle in proteinopathies involving extracellular or membrane-associated aggregates—such as α-synuclein—though the applicability of this principle appears to depend on whether the aggregating protein directly engages cell-surface receptor systems. This framework shifts the focus from aggregate burden to membrane organization and feedback topology as determinants of disease progression, and identifies the tau–Fyn feedback loop as a candidate therapeutic target in proteinopathies.

## Graphical abstract

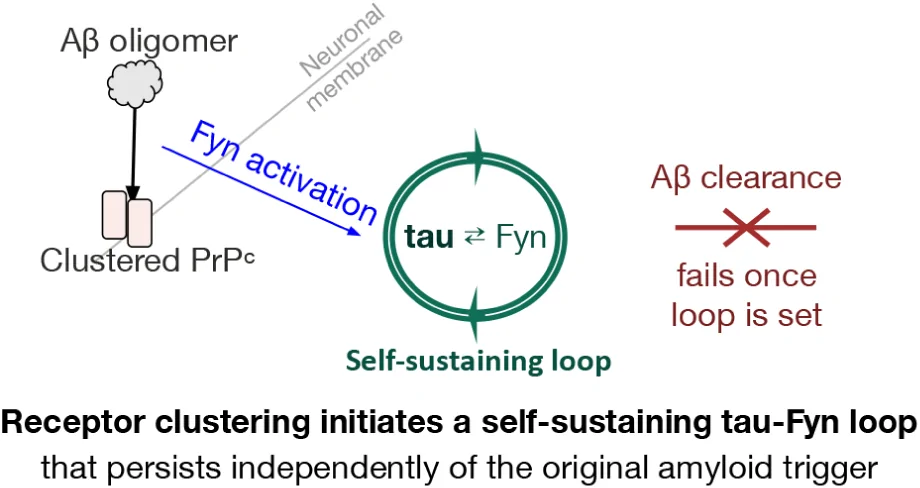

## Introduction

The failure of amyloid-targeted therapies in Alzheimer’s disease represents one of the most consequential puzzles in modern neuroscience. Despite decades of research and numerous clinical trials achieving measurable reductions in amyloid-β burden, cognitive decline has continued largely unabated in treated patients.^1^ This disconnection between aggregate clearance and clinical outcome suggests that, at least in established disease, the pathological process has become partially or fully independent of its initiating trigger, a possibility that current mechanistic frameworks do not adequately explain.

A central challenge in neurodegeneration research is to understand how extracellular protein aggregates initiate intracellular signaling programs that ultimately become self-sustaining. In Alzheimer’s disease, soluble oligomeric forms of amyloid-β, rather than insoluble fibrillar deposits, are increasingly recognized as the primary drivers of synaptic dysfunction.^2^ This distinction is important: it implies that toxicity arises from active molecular interactions at the cell surface, not from passive accumulation, and that the relevant pathological events may unfold rapidly upon oligomer engagement with neuronal receptors.^2^

Here, we propose a framework in which oligomeric protein assemblies act as multivalent scaffolds capable of cross-linking cell-surface receptors, inducing their spatial reorganization into signaling-competent membrane platforms. This clustering mechanism provides a physically grounded route by which extracellular aggregates initiate coordinated intracellular responses. Critically, we argue that downstream components of this signaling cascade, specifically the feedback interaction between hyperphosphorylated tau and Fyn kinase, can establish a self-amplifying loop that persists independently of the original aggregate signal.

This self-sustaining circuit offers a mechanistic explanation for the limited therapeutic window observed in antiamyloid strategies: interventions that successfully reduce oligomer burden may nonetheless fail if initiated after the feedback loop has been established. In this view, the relevant therapeutic target shifts from extracellular aggregates to the intracellular signaling topology that the aggregates set in motion.

We develop this argument using the amyloid-β/PrP^c^/mGluR5/Fyn/tau pathway as a representative example, and propose that clustering-driven feedback loops may represent a generalizable principle across proteinopathies. Rather than converging on a single toxic species, diverse protein aggregates may share a common capacity to initiate self-amplifying signaling states, a possibility with broad implications for how we conceptualize disease progression and therapeutic intervention in neurodegeneration.

### Conceptual Framework: Aggregates as Clustering-Driven Signaling Platforms

Protein aggregation is typically interpreted through the lens of misfolding and deposition. However, this Perspective does not fully account for the ability of soluble assemblies to elicit rapid and receptor-dependent cellular responses. An alternative view emerges when considering the physical properties of oligomeric aggregates: their multivalent architecture enables simultaneous engagement of multiple binding partners, a feature well known to drive clustering phenomena in membrane biology.^3^

Multivalent ligands are capable of cross-linking cell-surface receptors, thereby increasing their local density and promoting the formation of signaling-competent microdomains.^4^ In physiological contexts, similar mechanisms underlie processes such as immune receptor activation and growth factor signaling, where spatial organization at the membrane is a critical determinant of downstream responses. By analogy, extracellular protein aggregates may act as noncanonical ligands that reorganize receptor topology, rather than simply activating individual receptors in isolation.^5^

Within this framework, signaling does not arise solely from ligand–receptor affinity but from emergent properties of spatial organization, including receptor proximity, confinement within membrane nanodomains, and scaffold-assisted interactions. These features can lower activation thresholds for intracellular kinases and enable signal amplification through proximity-driven mechanisms. Importantly, such effects are expected to depend on aggregate properties such as size, valency, and structural heterogeneity, providing a potential explanation for the differential toxicity of oligomeric versus fibrillar species.^3^

This perspective suggests that extracellular aggregates function as architectural drivers of signaling, capable of converting molecular accumulation into organized, potentially pathological signaling platforms at the cell surface.

### A Clustering-Driven Signaling Platform Linking Extracellular Aβ to Intracellular Tau Dysregulation

A well-characterized example of this principle can be found in Alzheimer’s disease, where oligomeric amyloid-β assemblies engage receptor systems involving PrP^c^ and mGluR5, leading to activation of Fyn and downstream dysregulation of tau.^6,7^ Rather than representing a strictly linear cascade, this pathway can be interpreted as a clustering-driven signaling platform, in which spatial organization at the neuronal membrane enables signal initiation, amplification, and propagation (Figure 1). A detailed stepwise reconstruction of the underlying molecular interactions is provided in the Supporting Information 1.

**Figure 1:**
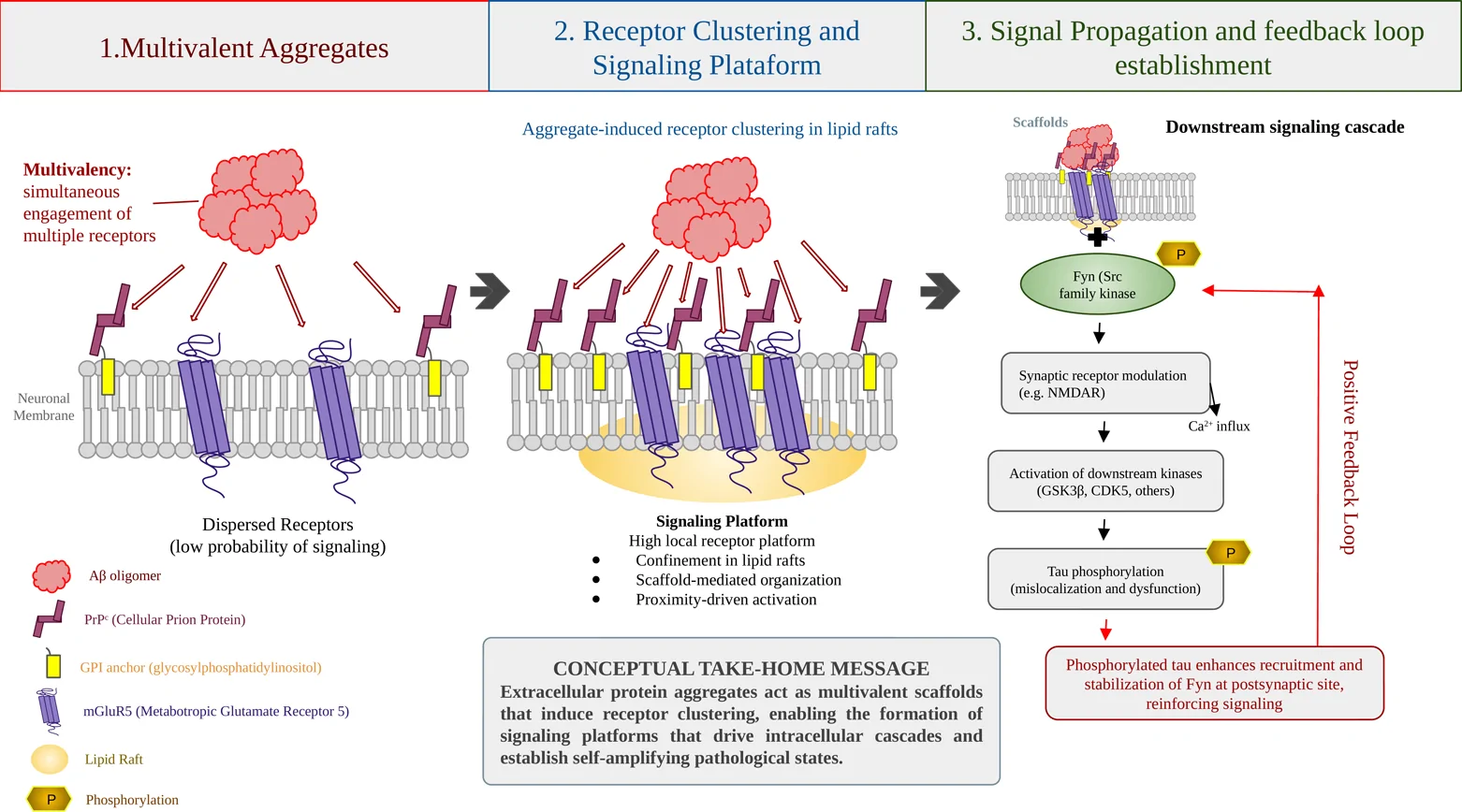
Protein aggregates as drivers of receptor clustering and self-sustaining pathological signaling. Schematic representation of the proposed framework in three stages. (1) Multivalent aggregates: Oligomeric amyloid-β assemblies engage multiple PrP^c^ molecules simultaneously at the neuronal surface, exploiting their intrinsic multivalency to interact with dispersed receptors under conditions of low signaling probability. (2) Receptor clustering and signaling platform assembly: Aggregate-induced cross-linking promotes nanoscale receptor clustering within lipid raft microdomains, facilitating PrP^c^-mGluR5 coupling and the scaffold-mediated recruitment and activation of Fyn kinase. Spatial confinement and proximity-driven interactions lower the activation threshold for downstream signaling. (3) Signal propagation and feedback loop establishment: Fyn activation leads to NMDAR phosphorylation, calcium influx, and activation of tau-regulating kinases including GSK3β and CDK5. Hyperphosphorylated tau redistributes to dendritic compartments, where it enhances Fyn recruitment and stabilization at postsynaptic sites, establishing a positive feedback loop that sustains and amplifies signaling independently of the initiating extracellular stimulus. This self-amplifying circuit provides a mechanistic basis for the progressive decoupling of synaptic dysfunction from amyloid burden.

### Aggregate-Induced Receptor Clustering

Oligomeric Aβ assemblies display intrinsic multivalency, allowing simultaneous engagement of multiple PrP^c^ molecules at the cell surface.^8^ Given that PrP^c^ is a glycosylphosphatidylinositol-anchored protein enriched in lipid raft microdomains, such interactions are expected to promote nanoscale receptor clustering and local increases in receptor density. This spatial reorganization provides a physical basis for signal initiation, independent of classical ligand–receptor activation paradigms, by increasing the probability of productive receptor–receptor and receptor–effector interactions.^5^

### Assembly of a Membrane-Associated Signaling Platform

Because PrP^c^ lacks an intracellular domain, signal transduction depends on its coupling to transmembrane partners, notably mGluR5.^6,7^ Within Aβ-induced clusters, this interaction enables the formation of higher-order signaling complexes in which receptor proximity and confinement within membrane nanodomains facilitate downstream activation events. In this context, recruitment and activation of Fyn can be understood as a proximity-driven process, supported by scaffold-mediated interactions and local enrichment of signaling components.^9^ Rather than a strictly sequential pathway, kinase activation emerges from the spatial co-organization of receptors, scaffolds, and enzymes within confined membrane regions.

### Signal Propagation and Pathological Feedback

Downstream of Fyn activation, phosphorylation of NMDA receptor subunits, particularly GluN2B, enhances calcium influx and creates a cellular environment that favors activation of tau-regulating kinases, including GSK3β and CDK5.^10–12^ These kinases phosphorylate tau at multiple epitopes associated with pathological redistribution, promoting its translocation from axonal to dendritic compartments. This relocalization is not merely a passive consequence of hyperphosphorylation: it positions tau at postsynaptic sites where Fyn is actively engaged with receptor complexes, establishing the conditions for a positive feedback interaction.

Critically, dendritic tau does not simply accumulate as an inert substrate.^13^ Hyperphosphorylated tau enhances the recruitment and stabilization of Fyn at postsynaptic densities, thereby sustaining and amplifying the very kinase activity responsible for its own phosphorylation.^14,15^ This constitutes a self-reinforcing loop in which tau pathology and Fyn activation become mutually dependent, each driving the other forward, independent of upstream receptor engagement.

The implications of this feedback architecture are significant. Once established, the loop does not require continued input from extracellular amyloid-β oligomers to remain active. The signaling state initiated by aggregate-driven receptor clustering can, in principle, persist and amplify even after the initiating stimulus has been removed. This predicts the existence of a pathological threshold, a point beyond which the system becomes self-sustaining and resistant to interventions targeting the original trigger.

This threshold concept provides a direct mechanistic explanation for one of the most consequential observations in Alzheimer’s disease research: the consistent failure of antiamyloid therapies to halt cognitive decline in patients with established disease.^1^ Clinical trials achieving significant reductions in amyloid burden, including those targeting soluble oligomeric species, have largely failed to produce corresponding cognitive benefits. Within the present framework, this outcome is expected if treatment is initiated after the tau-Fyn feedback loop has already been consolidated. At that stage, aggregate clearance addresses the initiating event but leaves the self-amplifying intracellular circuit intact.

This interpretation reframes the therapeutic window problem in neurodegeneration not as a question of aggregate load, but as a question of feedback loop consolidation. The relevant variable is not how much amyloid is present, but whether the downstream signaling topology has reached a self-sustaining state. Accordingly, the framework predicts that early intervention, prior to feedback loop establishment, should produce substantially greater benefit than late intervention, even if the aggregate clearance is equivalent in both cases. Conversely, therapeutic strategies targeting components of the feedback loop itself, such as Fyn kinase activity or tau-dependent postsynaptic scaffolding, may retain efficacy at later disease stages, when amyloid-directed approaches have already lost their window of opportunity.

It is important to note that this feedback mechanism may not be unique to Alzheimer’s disease. The general principle, whereby an aggregate-initiated kinase activation event creates conditions for a self-sustaining intracellular loop, may operate across diverse proteinopathies. Wherever protein aggregates engage receptor systems capable of activating kinases that phosphorylate cytoskeletal or scaffolding proteins, the possibility of analogous feedback architectures should be considered. This generalization is developed further in the following section.

### Generalization: Extracellular Aggregates as Universal Drivers of Receptor Clustering

The clustering-driven signaling model described above may extend beyond Alzheimer’s disease, though the strength of supporting evidence varies considerably across proteinopathies and this variation must be made explicit.

The most direct parallel is found in α-synucleinopathies. Oligomeric and fibrillar α-synuclein assemblies have been shown to cluster at the neuronal plasma membrane and to cocluster with membrane proteins such as the α3 subunit of the Na^+^/K^+^ -ATPase, leading to its functional redistribution and impaired ion transport. Distinct fibrillar α-synuclein polymorphs have further been shown to bind and cluster differentially at the plasma membrane, producing polymorph-dependent redistribution of synaptic glutamate receptors, including GluN2B-containing NMDA receptors and GluA2-containing AMPA receptors.^16,17^ These findings provide a relatively close experimental parallel to the receptor clustering mechanism proposed here, supporting the possibility that analogous clustering-driven signaling platforms operate in Parkinson’s disease and related synucleinopathies.

The case for huntingtin-related disorders is comparatively weaker and qualitatively distinct. Available evidence indicates that mutant huntingtin alters the trafficking and synaptic localization of NMDA receptors through intracellular protein–protein interactions, including disrupted coupling to PSD-95 and altered receptor palmitoylation. While this demonstrates that huntingtin pathology can reorganize receptor distribution at the membrane, the underlying mechanism is fundamentally different from the extracellular, multivalency-driven clustering proposed for amyloid-β and α-synuclein: it reflects intracellular trafficking dysregulation by a predominantly nuclear and cytoplasmic protein, rather than direct multivalent engagement of cell-surface receptors by an extracellular aggregate.^18,19^ We therefore regard the extension of this framework to Huntington’s disease as a more distant and speculative analogy, useful primarily as a contrast that helps clarify the boundary conditions of the proposed mechanism.

TDP-43 proteinopathies illustrate this boundary more sharply still. TDP-43 pathology is characterized predominantly by nuclear depletion, cytoplasmic mislocalization, and aggregation within stress granules, with toxicity arising largely from disrupted RNA processing and loss of nuclear function rather than from direct engagement of cell-surface receptor systems.^20^ To our knowledge, no evidence currently demonstrates that extracellular or membrane-proximal TDP-43 assemblies induce receptor clustering analogous to that described for amyloid-β or α-synuclein. We therefore do not extend the clustering-driven feedback model to TDP-43 proteinopathies in its current form. We note this explicitly because we consider it important that a generalizable framework also specifies where it is not expected to apply: TDP-43 pathology may instead represent an informative negative case, illustrating that aggregate-induced membrane receptor clustering is unlikely to be a universal feature of proteinopathies, but rather a mechanism specific to aggregates that engage cell-surface receptor systems as part of their pathogenic process.

Taken together, these comparisons suggest that the clustering-driven signaling principle proposed here is best understood not as a universal mechanism applicable to all protein aggregation disorders, but as a framework whose applicability depends on whether the aggregating protein engages extracellular or cell-surface receptor systems. This distinction itself constitutes a testable boundary condition: proteinopathies involving predominantly intracellular, nuclear, or cytoplasmic aggregation, such as TDP-43, would not be expected to exhibit the same clustering-dependent feedback dynamics as those involving extracellular or membrane-associated aggregates, such as amyloid-β and α-synuclein.

### Implications and Testable Predictions

The framework proposed here generates a set of concrete, experimentally addressable predictions that distinguish it from purely descriptive accounts of amyloid-β toxicity. These predictions span multiple levels of analysis, from membrane organization to clinical outcome, and collectively define a shift in experimental focus, from aggregate burden to signaling topology and feedback loop dynamics.

Prediction 1: Aggregate removal should fail to rescue synaptic function after feedback-loop consolidation;

The most direct prediction of the framework concerns the therapeutic window for antiamyloid interventions. If the tau-Fyn feedback loop can become self-sustaining, then removal of amyloid-β oligomers after loop consolidation should fail to reverse synaptic dysfunction, even when aggregate clearance is substantial. This prediction is broadly consistent with the outcomes of major clinical trials in Alzheimer’s disease, including those achieving significant reductions in amyloid burden.^1^ However, it goes beyond post hoc rationalization: it predicts a specific stage dependence of treatment efficacy, in which the same intervention produces markedly different outcomes depending on whether it is administered before or after feedback loop establishment. This prediction is testable in animal models by combining temporally controlled oligomer clearance with quantitative assessment of tau-Fyn colocalization and postsynaptic Fyn activity as proxies for loop consolidation.

Prediction 2: Disrupting receptor clustering should attenuate downstream signaling independently of aggregate removal.

If extracellular oligomers act primarily by cross-linking receptors and inducing nanoscale clustering, then interventions that interfere with receptor mobility or membrane compartmentalization should reduce signaling output even in the continued presence of aggregates. This prediction is experimentally accessible through manipulation of lipid raft integrity, receptor lateral diffusion, or PrP^c^-mGluR5 coupling. Critically, this approach would decouple the question of aggregate burden from the question of signaling competence, allowing for direct testing of the clustering hypothesis independently of amyloid clearance.

Prediction 3: Aggregate valency should determine signaling potency.

The model predicts that oligomeric assemblies, which present multiple accessible binding interfaces, should be more effective at inducing receptor clustering than larger, structurally constrained fibrils. This predicts a nonmonotonic relationship between aggregate size and biological activity, in which intermediate-sized oligomers, with high effective valency and structural flexibility, represent the most potent signaling inducers. Controlled engineering of multivalent assemblies with defined size and valency could directly test this relationship, with signaling outputs measured by Fyn activation, Tau phosphorylation, and downstream calcium dynamics.

Prediction 4: Partial aggregate disruption may paradoxically enhance signaling.

A nonintuitive consequence of the valency model is that fragmentation of larger aggregates into smaller oligomeric species could increase effective valency and enhance receptor cross-linking, potentially amplifying rather than suppressing signaling. This predicts that therapeutic strategies aimed at disaggregating mature fibrils without achieving complete clearance could produce paradoxical increases in synaptic toxicity. This prediction has direct implications for the interpretation of disaggregation-based therapeutic approaches and suggests that the aggregate size distribution, not total burden, should be monitored as a primary outcome variable.

Prediction 5: Targeting the tau-Fyn feedback loop should retain efficacy at late disease stages.

In contrast to antiamyloid strategies, interventions targeting the feedback loop itself, including Fyn kinase inhibitors, tau phosphorylation blockers, or disruptors of tau-dependent postsynaptic scaffolding, should retain therapeutic efficacy even after the loop has been consolidated and the initiating amyloid signal is no longer required. This prediction defines a complementary therapeutic strategy for patients who have passed the window of opportunity for amyloid-directed intervention and suggests that combination approaches targeting both the initiating clustering mechanism and the downstream feedback topology may outperform single-target strategies across a broader range of disease stages.

Prediction 6: Cellular vulnerability should correlate with receptor landscape organization.

The framework predicts that cell-type- and region-specific susceptibility to aggregate-induced signaling is determined not only by aggregate exposure but also by the organization of the receptor landscape, including receptor density, lateral mobility, and scaffold availability. Neurons with higher PrP^c^ surface density, greater lipid raft compartmentalization, or more abundant postsynaptic Fyn scaffolding should exhibit lower clustering thresholds and greater signaling amplification in response to equivalent oligomer exposure. This prediction offers a potential explanation for the selective neurodegeneration observed across proteinopathies and is testable through the correlative analysis of receptor organization and vulnerability across neuronal subtypes.

ESTABLISHED EVIDENCE AND INTERPRETIVE EXTRAPOLATION

The framework proposed here integrates findings from the established experimental literature with novel interpretive claims, and it is important to distinguish explicitly between these two categories.

The molecular components of the central pathway discussed here are supported by direct experimental evidence. The identification of PrP^c^ as a high-affinity receptor for oligomeric amyloid-β, its functional coupling to mGluR5, and the resulting activation of Fyn kinase have been demonstrated in independent studies using biochemical, electrophysiological, and *in vivo* approaches.^6,7^ Similarly, the role of Fyn in promoting tau phosphorylation and the capacity of pathologically redistributed tau to enhance postsynaptic Fyn localization are supported by direct experimental observation.^13^ The preferential synaptotoxicity of soluble oligomeric Aβ relative to fibrillar deposits is likewise well established in the literature.^3^

In contrast, not directly demonstrated by existing data, and constituting the interpretive contribution of this outlook, is the proposal that these established molecular events collectively assemble into a clustering-driven signaling platform whose behavior is governed by emergent properties of spatial organization, and that the tau-Fyn interaction constitutes a self-sustaining feedback loop capable of persisting independently of continued amyloid-β engagement. While each individual link in this chain is empirically supported, the claim that the system as a whole reaches a self-sustaining state, and that this transition explains the limited efficacy of amyloid-targeted therapies in established disease, is an interpretive extrapolation. It is consistent with, but not directly tested by, existing data, and is offered here as a falsifiable hypothesis rather than an established conclusion. Direct experimental support for this claim would require demonstrating that tau–Fyn signaling persists, or even intensifies, following near-complete clearance of extracellular amyloid-β oligomers, an experiment that, to our knowledge, has not yet been performed under conditions designed specifically to test this prediction.

The generalization of this framework to other proteinopathies is offered at a still greater distance from direct evidence, and its strength varies considerably depending on the protein considered. For α-synuclein, direct experimental evidence of membrane clustering and receptor coredistribution provides reasonable support for an analogous mechanism, though whether this clustering establishes a self-sustaining feedback loop comparable to tau–Fyn has not been directly tested. For huntingtin, existing evidence concerns intracellular receptor trafficking rather than extracellular multivalent clustering, making this extension considerably more speculative. For TDP-43, no evidence currently supports extracellular receptor clustering as a relevant mechanism, and we do not extend the framework to this proteinopathy. This generalization should therefore be understood as a hypothesis of variable strength across proteinopathies, intended to guide future investigation rather than as a uniform conclusion supported by equivalent cross-disease evidence.

We consider this distinction important not as a caveat that weakens the framework but as a necessary clarification of its current evidentiary status. The value of the proposed model lies in its capacity to generate specific, testable predictions; its validity as a mechanistic account of disease progression remains to be established through the experiments outlined in the preceding section.

## Conclusion

We propose that extracellular protein aggregation can be understood not solely as a process of molecular deposition but as a driver of spatially organized signaling at the cell surface. By acting as multivalent scaffolds, protein aggregates may induce receptor clustering and promote the emergence of signaling platforms capable of coupling extracellular events to intracellular responses. Within this framework, the pathway linking amyloid-β to PrP^c^, mGluR5, Fyn, and tau can be interpreted as a representative instance of a broader organizational principle, in which signaling emerges from receptor proximity, membrane confinement, and scaffold-mediated interactions rather than from strictly linear pathways.

This perspective shifts attention from the biochemical identity of aggregates to their architectural and physical properties, including valency, geometry, and capacity to reorganize membrane topology. Such a shift may help reconcile diverse observations across proteinopathies and suggest that convergent pathological outcomes may arise from shared mechanisms of clustering-driven signaling.

More broadly, this work highlights the importance of spatial organization as a determinant of cellular signaling in disease. By the integration of concepts from protein aggregation, membrane biology, and signaling dynamics, the proposed framework provides a basis for reinterpreting extracellular aggregates as active modulators of cellular behavior. Future efforts aimed at quantifying and manipulating receptor clustering, membrane organization, and signaling topology may not only refine our understanding of neurodegeneration but also reveal new avenues for therapeutic interventions.

## Supplementary Material





## Notes

Supporting Information: Stepwise molecular reconstruction of the amyloid-β/PrP^c^/mGluR5/Fyn/tau signaling pathway, including mechanistic details of receptor clustering, Fyn structural activation, tau hyperphosphorylation, and feedback loop establishment; schematic figure illustrating the complete signaling cascade from extracellular aggregate engagement to intracellular tau–Fyn feedback (PDF)
