# Supplementary material for: Protein Aggregates as Drivers of Receptor Clustering and Pathological Signaling: A Framework Linking Extracellular Amyloid‑β to Tau Dysregulation
Source: ACS Chem Neurosci. 2026 Aug 20;17(17):3150–5. doi: 10.1021/acschemneuro.6c00331 (PMC13543654; doi:10.1021/acschemneuro.6c00331)
Supplement: Supplementary file 1 [file cn6c00331_si_001.pdf]

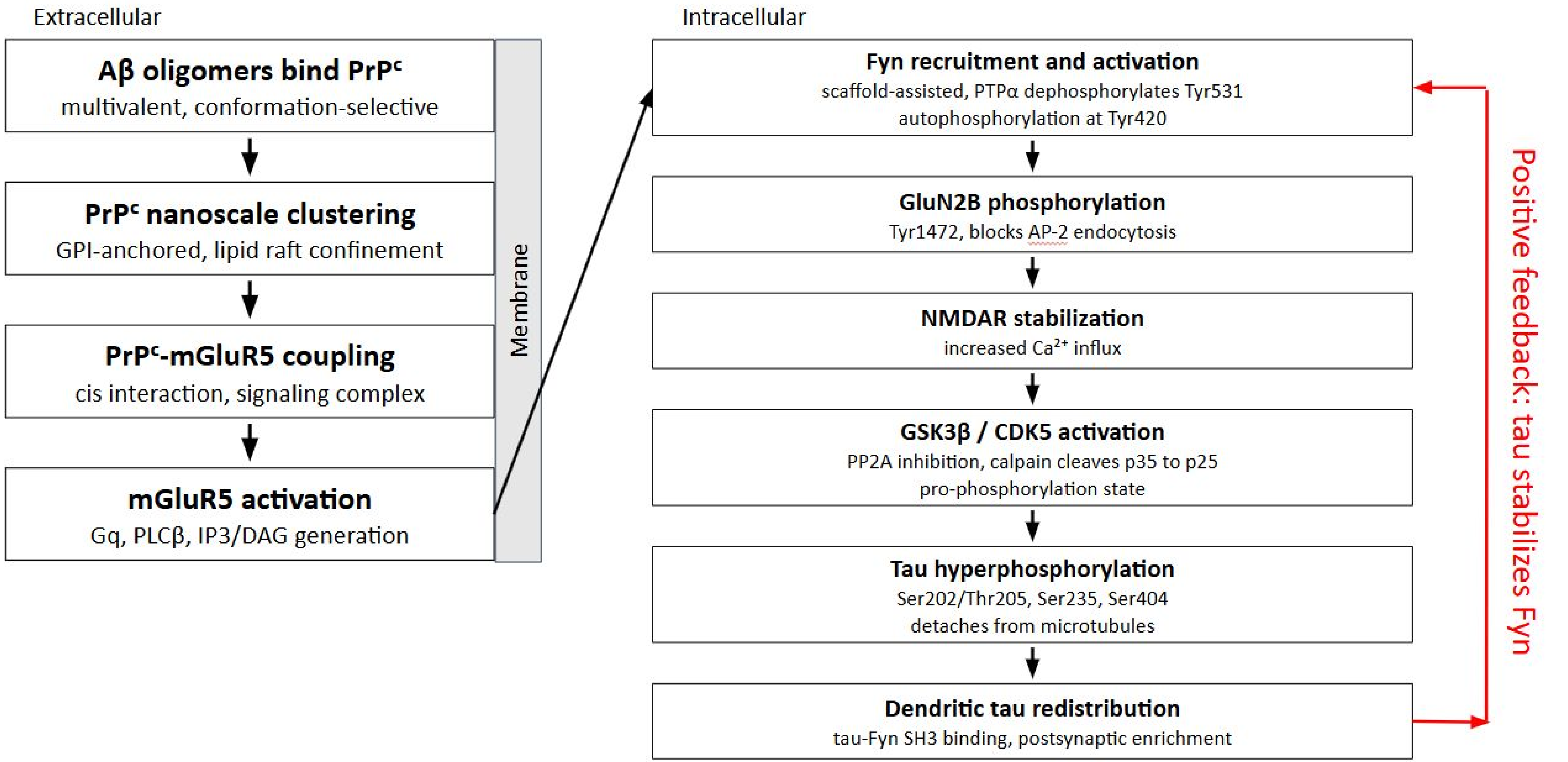

Figure A. Receptor clustering and the tau–Fyn feedback loop linking extracellular amyloid-β to intracellular signaling dysregulation.

Schematic representation of the proposed clustering-driven signaling cascade. Extracellular amyloid-β oligomers engage and cluster GPI-anchored PrP^c at the neuronal membrane, promoting its functional coupling to mGluR5 and downstream activation of Fyn kinase. Intracellularly, Fyn phosphorylates GluN2B, stabilizing NMDA receptors and increasing Ca^{2+} influx, which activates GSK3β and CDK5 through PP2A inhibition and calpain-mediated p35-to-p25 cleavage. The resulting tau hyperphosphorylation promotes its redistribution to dendritic compartments, where tau enhances Fyn recruitment and stabilization at the postsynaptic density, establishing a self-sustaining positive feedback loop (red) that perpetuates signaling independently of continued amyloid-β engagement.
