# Supplementary material for: Protein Aggregates as Drivers of Receptor Clustering and Pathological Signaling: A Framework Linking Extracellular Amyloid‑β to Tau Dysregulation
Source: ACS Chem Neurosci. 2026 Aug 20;17(17):3150–5. doi: 10.1021/acschemneuro.6c00331 (PMC13543654; doi:10.1021/acschemneuro.6c00331)
Supplement: Supplementary file 2 [file cn6c00331_si_002.pdf]

**Proposed mechanistic cascade linking Aβ oligomers to tau pathology via $PrP^c$–mGluR5–Fyn signaling - Figure A**

**1. Aβ - $PrP^c$ - linking and clustering**

Extracellular oligomeric amyloid-β (Aβ) assemblies have been shown to bind the cellular prion protein ($PrP^c$) with high affinity, mediating key aspects of synaptic dysfunction associated with Alzheimer's disease (1). This interaction is conformationally selective, preferentially involving oligomeric rather than monomeric Aβ species, and is mediated by the intrinsically disordered N-terminal domain of $PrP^c$, particularly residues encompassing positions ~23–27 and ~95–110 (2,3). These regions contain positively charged and hydrophobic elements that contribute to a composite binding interface, consistent with electrostatic and hydrophobic interactions that favor β-sheet–rich Aβ conformations (2,4).

Given the multivalent nature of Aβ oligomers, which present multiple binding interfaces, their interaction with $PrP^c$ is consistent with the formation of higher-order receptor–ligand assemblies that may enhance binding avidity (2). $PrP^c$ is a glycosylphosphatidylinositol (GPI)-anchored protein localized within cholesterol-rich membrane microdomains (lipid rafts), where it can undergo lateral organization and clustering (2,5). Within this membrane context, engagement of $PrP^c$ by Aβ oligomers may promote nanoscale clustering of receptor complexes, thereby increasing local receptor density and facilitating downstream signaling processes associated with synaptic toxicity. It should be noted, however, that the precise molecular mechanism linking mGluR5 activation to Fyn recruitment remains incompletely defined, and may involve indirect scaffold-mediated interactions or β-arrestin-dependent pathways rather than a direct receptor-kinase coupling.

**2. $PrP^c$ - mGluR5 - signal transduction**

Given that the cellular prion protein ($PrP^c$) lacks a transmembrane and intracellular signaling domain, its ability to transduce extracellular signals depends on lateral (cis) interactions with transmembrane co-receptors. In this context, $PrP^c$ has been shown to functionally couple with the metabotropic glutamate receptor 5 (mGluR5), a class C G protein-coupled receptor enriched in cholesterol-rich membrane microdomains (6,7). This interaction enables $PrP^c$-bound amyloid-β (Aβ) oligomers to engage intracellular signaling pathways via mGluR5.

Experimental evidence indicates that Aβ oligomers promote the formation of $PrP^c$–mGluR5 signaling complexes at the neuronal surface, facilitating receptor clustering and enhancing downstream signaling (6). Within this framework, Aβ-induced nanoscale organization of $PrP^c$ is proposed to promote the recruitment and stabilization of mGluR5 within membrane nanodomains, potentially through transient extracellular interactions and scaffold-mediated mechanisms (7). This spatial reorganization may influence the conformational dynamics of mGluR5, enabling receptor activation in a ligand-independent or sensitized manner.

Upon activation, mGluR5 couples to Gq proteins, leading to the activation of phospholipase Cβ (PLCβ), hydrolysis of phosphatidylinositol 4,5-bisphosphate ($PIP_2$), and the generation of inositol 1,4,5-trisphosphate ($IP_3$) and diacylglycerol (DAG), ultimately resulting in intracellular $Ca^{2+}$ mobilization (8). Through this mechanism, the $PrP^c$-mGluR5 complex provides a functional bridge between extracellular Aβ oligomers and intracellular calcium-dependent signaling pathways implicated in synaptic dysfunction.

### 3. **mGluR5 - Fyn recruitment**

Concomitantly, activation of the metabotropic glutamate receptor 5 (mGluR5) within amyloid-β (Aβ)-induced membrane nanodomains is proposed to facilitate the spatial recruitment and activation of the Src-family tyrosine kinase Fyn through a proximity-driven, scaffold-assisted mechanism rather

than a strictly linear signaling cascade. Fyn is a non-receptor tyrosine kinase constitutively associated with the inner leaflet of the plasma membrane via N-terminal myristoylation and palmitoylation, which promote its localization to membrane microdomains enriched in signaling proteins (9,10).

In the context of Alzheimer's disease, Aβ oligomer binding to the cellular prion protein ($PrP^c$) has been shown to trigger Fyn activation, linking extracellular amyloid signaling to intracellular kinase pathways (11,12). Moreover, $PrP^c$ has been reported to participate in membrane-associated signaling complexes capable of recruiting Fyn through scaffold-mediated interactions (13). Within this framework, the clustering of $PrP^c$ and mGluR5 induced by Aβ oligomers is likely to promote local enrichment of Fyn in proximity to activated receptor complexes, thereby increasing the probability of kinase activation through spatial confinement and enhanced protein-protein interactions. This model is consistent with emerging evidence indicating that Aβ-$PrP^c$ signaling leads to Fyn activation and downstream synaptic dysfunction via NMDA receptor modulation (11,12).

**4. Tau / PSD-95 - Fyn synaptic organization**

Simultaneously, cytoskeletal and postsynaptic density scaffolds facilitate the physical engagement and spatial organization of Fyn within synaptic signaling complexes. In particular, tau has been shown to directly interact with Fyn through proline-rich PXXP motifs located in its projection domain, which bind to the SH3 domain of Fyn (14,15). This interaction promotes the targeting of Fyn to the postsynaptic compartment and is consistent with mechanisms by which SH3-binding ligands can disrupt intramolecular SH3-linker interactions that contribute to the autoinhibited conformation of Src-family kinases (16).

In addition to tau, scaffold proteins such as PSD-95 play a central role in organizing postsynaptic signaling complexes by anchoring Fyn in proximity to glutamate receptors, including NMDA receptors (17). These scaffold-mediated interactions contribute to the stabilization of Fyn within the

postsynaptic density, increasing its local residence time and facilitating efficient signal propagation. Within this framework, tau and postsynaptic scaffold proteins act cooperatively to regulate the spatial localization and functional availability of Fyn in synaptic compartments.

## 5. Structural activation of Fyn

Relief of Fyn autoinhibition is initiated by dephosphorylation of the inhibitory C-terminal tyrosine residue (Tyr531 in human Fyn), a regulatory mechanism conserved across Src-family kinases (16,18). This residue participates in an intramolecular interaction with the SH2 domain that stabilizes the kinase in a closed, inactive conformation. Dephosphorylation of Tyr531, mediated by membrane-associated protein tyrosine phosphatases such as PTPα, disrupts this SH2–pTyr interaction and promotes a conformational transition toward an open, activation-competent state (19,20).

PTPα is localized to the plasma membrane and has been reported to associate with lipid raft microdomains, where it can regulate Src-family kinase activity (19,20). Within this context, membrane reorganization induced by amyloid-β (Aβ) oligomers may enhance the spatial proximity between Fyn and its regulatory phosphatases, facilitating access to Tyr531 and promoting kinase activation.

In parallel, engagement of the SH3 domain by proline-rich ligands, including tau, may further destabilize the autoinhibited conformation by disrupting intramolecular SH3-linker interactions, thereby favoring the transition to an active kinase state (16,21). Together, these mechanisms provide a coordinated structural basis for Fyn activation through combined SH2 and SH3 domain regulation within membrane-associated signaling complexes.

## 6. Fyn - NMDA receptor - $Ca^{2+}$

In this permissive conformation, Fyn undergoes trans-autophosphorylation at the activation loop tyrosine residue (Tyr420), a conserved regulatory mechanism among Src-family kinases that stabilizes the active site and fully enables catalytic activity (16,18). Once activated, Fyn phosphorylates the intracellular C-terminal domain of the NMDA receptor subunit GluN2B at Tyr1472, a critical regulatory site involved in controlling receptor trafficking and synaptic localization (22,23).

Phosphorylation at Tyr1472 disrupts the interaction between GluN2B and the AP-2 endocytic adaptor complex, thereby inhibiting clathrin-mediated internalization of NMDA receptors (23). As a result, NMDA receptors are stabilized at the postsynaptic membrane, leading to increased synaptic receptor density and enhanced calcium permeability upon activation. This Fyn-dependent modulation of NMDA receptor trafficking provides a direct mechanistic link between upstream receptor signaling and downstream calcium-mediated synaptic dysfunction.

**7. $Ca^{2+}$ - PP2A / GSK3β / CDK5**

Elevated intracellular $Ca^{2+}$ levels downstream of NMDA receptor overactivation engage multiple signaling pathways that collectively shift the intracellular environment toward a pro-phosphorylation state (24,25). Among these, the serine/threonine phosphatase PP2A plays a central role in tau regulation and is impaired in Alzheimer's disease (26). Aβ has been shown to disrupt PP2A activity through altered methylation (LCMT-1) and increased expression of endogenous inhibitors such as I2PP2A.

Reduced PP2A activity leads to decreased Akt activity, which in turn relieves inhibitory phosphorylation of glycogen synthase kinase-3β (GSK3β) at Ser9, resulting in increased kinase activity (27,28). Simultaneously, $Ca^{2+}$-dependent activation of calpain promotes cleavage of p35 into p25, resulting in sustained CDK5 activation (29). Notably, unlike the transient kinase activation

associated with p35, the p25-CDK5 complex exhibits prolonged stability due to the extended half-life of p25, thereby sustaining pathological substrate phosphorylation beyond physiological limits. Together, these converging pathways establish a kinase environment that favors pathological tau phosphorylation.

**8. Tau hyperphosphorylation and dendritic redistribution of Fyn**

Hyperactivation of glycogen synthase kinase-3β (GSK3β) and cyclin-dependent kinase 5 (CDK5) leads to phosphorylation of tau at multiple pathological epitopes, including Ser202/Thr205, Ser235, and Ser404, which are commonly associated with neurofibrillary pathology in Alzheimer's disease (30,31). These phosphorylation events reduce tau affinity for microtubules, impairing its stabilizing function and promoting its detachment and redistribution from axonal compartments to the somatodendritic domain (32–34). The precise mechanisms underlying this compartmental redistribution remain debated, with proposed contributions from disruption of axon initial segment diffusion barriers, impaired microtubule-based transport, and passive diffusion following cytoskeletal destabilization.

In addition to its role as a microtubule-associated protein, tau directly interacts with the Src-family kinase Fyn through proline-rich PXXP motifs that bind to the SH3 domain of Fyn (21). Under physiological conditions, this interaction contributes to the trafficking of Fyn along microtubules and supports its localization to the postsynaptic density, where it participates in synaptic signaling complexes (15).

**9. Tau-Fyn positive feedback loop amplifies synaptic dysfunction**

However, upon hyperphosphorylation and detachment from microtubules, tau accumulates in dendritic compartments, a redistribution that has been strongly associated with synaptic dysfunction in Alzheimer's disease (15,35). This mislocalization enhances the local recruitment and retention of

Fyn at postsynaptic sites, as tau functions as a targeting scaffold that facilitates Fyn positioning within dendritic compartments (15,36).

The resulting enrichment of Fyn at synapses promotes increased phosphorylation of the NMDA receptor subunit GluN2B, contributing to receptor stabilization and enhanced calcium influx (22,23). This amplification of NMDA receptor signaling reinforces downstream kinase activation, including pathways involving GSK3β and CDK5, thereby further promoting tau phosphorylation.

Within this framework, tau acts not only as a downstream substrate of kinase signaling but also as a spatial organizer of synaptic signaling complexes. This dual role establishes a self-reinforcing pathological feedback loop in which tau mislocalization enhances Fyn-dependent signaling, which in turn exacerbates tau hyperphosphorylation and synaptic dysfunction.

**Mechanistic predictions and experimental implications**

Collectively, the proposed model generates several testable predictions that can be experimentally addressed to validate the mechanistic links between extracellular amyloid-β signaling and intracellular tau pathology.

First, disruption of the interaction between tau and Fyn, such as through mutation of tau proline-rich PXXP motifs or pharmacological inhibition of SH3 domain binding, is expected to impair the dendritic localization of Fyn and attenuate amyloid-β–induced stabilization of NMDA receptors at the postsynaptic membrane.

Second, interference with $PrP^c$ clustering or disruption of lipid raft integrity is predicted to reduce Fyn activation downstream of amyloid-β oligomers (25), supporting a proximity-dependent mechanism of signal transduction. In parallel, pharmacological inhibition of mGluR5 is expected to

suppress intracellular calcium signaling and downstream kinase activation, even in the presence of intact $PrP^c$, confirming its role as a critical transducer in this pathway.

At the intracellular level, restoration of PP2A activity, through modulation of LCMT-1 or inhibition of endogenous PP2A inhibitors such as I2PP2A, is expected to reduce tau phosphorylation independently of upstream receptor activation, identifying PP2A as a central convergence point in the signaling cascade. Similarly, inhibition of calpain activity is predicted to prevent the conversion of p35 to p25, thereby limiting CDK5 hyperactivation and reducing pathological tau phosphorylation.

Importantly, the model predicts that tau mislocalization to dendritic compartments may be sufficient to enhance synaptic Fyn recruitment and increase NMDA receptor phosphorylation, even in the absence of exogenous amyloid-β, supporting the existence of a self-reinforcing pathological feedback loop.

Finally, combined targeting of both upstream signaling components (such as Fyn or mGluR5) and downstream tau kinases (including GSK3β and CDK5) is expected to produce synergistic effects in reducing tau pathology, reflecting the integrated and multilevel nature of the proposed signaling network.

Together, these predictions provide a framework for experimental validation and highlight potential therapeutic intervention points within the $PrP^c$–mGluR5–Fyn–tau signaling axis.

**Limitations and conflicting evidence**

While the proposed signaling cascade is grounded in published experimental evidence, several lines of conflicting data warrant explicit acknowledgment. First, studies employing $PrP^c$ knockout models have demonstrated that Aβ-induced synaptic toxicity is not entirely abolished in the absence of $PrP^c$ expression, suggesting that parallel receptor-mediated pathways, including LilrB2 and other synaptic

proteins, may contribute independently to amyloid-driven neuronal dysfunction. This observation implies that $PrP^c$, while a relevant transducer, may not be strictly necessary for all manifestations of Aβ toxicity, and that the proposed cascade may represent one of several converging pathogenic routes rather than a singular obligatory pathway.

Second, the role of mGluR5 in Alzheimer's disease models has yielded inconsistent results. Pharmacological antagonism of mGluR5, including with compounds such as MPEP and MTEP, has produced variable outcomes across different transgenic mouse models, with some studies reporting rescue of synaptic deficits and others showing limited or no effect on amyloid-associated pathology. These discrepancies may reflect differences in disease stage, model background, or the relative contribution of mGluR5-dependent signaling across experimental contexts, but they underscore the need for caution in generalizing findings across systems.

Third, the mechanistic link between mGluR5 activation and Fyn recruitment, as discussed in section 3, remains incompletely characterized at the molecular level. Although proximity-driven and scaffold-mediated mechanisms are consistent with available data, direct experimental evidence for mGluR5-dependent Fyn activation in the context of Aβ signaling is limited, and indirect or compensatory pathways cannot be excluded.

Fourth, the evidence linking tau mislocalization to enhanced synaptic Fyn recruitment relies substantially on the seminal work of Ittner and colleagues (2010). Independent replication of this specific mechanism in distinct experimental systems remains limited, and the generalizability of this finding across neuronal subtypes and disease stages has not been fully established.

Finally, the proposed cascade was assembled from findings obtained across heterogeneous experimental systems, including primary neuronal cultures, transgenic rodent models, and postmortem human tissue. The extent to which this integrated pathway operates as a unified signaling

axis in the human brain, and whether its relative contribution varies across disease stages or brain regions, remains to be determined. These considerations highlight the importance of experimental validation using unified model systems before therapeutic implications of the proposed framework can be fully assessed.

The strength of this framework lies in its ability to generate testable predictions and identify key points of therapeutic intervention. Targeting receptor-level interactions, modulating Fyn activity, restoring PP2A function, or disrupting the tau–Fyn feedback loop all emerge as potential strategies to interrupt the progression of synaptic pathology. Importantly, the model suggests that combinatorial approaches targeting multiple levels of the signaling cascade may be more effective than single-target interventions.

**AI USE**

Claude, version sonnet 4.6, was used to check for inconsistencies in the described route; it pointed out the following 4 issues (which were corrected later). For transparency: *"1. The mGluR5 → Fyn connection (section 3) is the weakest link. You yourself use cautious language ("is proposed to", "likely to"), which is honest, but the mechanism by which activated mGluR5 recruits Fyn is not fully elucidated in the literature. It is worth explicitly acknowledging that this step may be indirect—possibly mediated by β-arrestin or by microdomain rearrangement, for example. 2. PP2A (section 7) — minor typographical error. You wrote "altered mthylation" — the "e" is missing in methylation. Furthermore, the logic PP2A → Akt → GSK3β is valid, but it's a secondary pathway. Direct inhibition of GSK3β by Akt (pSer9) and activation of GSK3β by $Ca^{2+}$/CaMKII could be integrated to strengthen this section. 3. CDK5/p25 deserves more context. The cleavage of p35 → p25 by calpain is well documented (Patrick 1999), but it would be useful to mention that p25 extends the half-life of CDK5 activation,*

*distinguishing it from physiological activation—this is precisely the pathological aspect. 4. The axon → somatodendritic transition (section 8) You describe hyperphosphorylation well, but the mechanism by which tau leaves the axon and accumulates in the dendrite is still debated (does it involve retrograde transport? passive diffusion? collapse of axonal barriers?). A sentence acknowledging this would strengthen the credibility of the text."*

**References**


1. Laurén J, Gimbel DA, Nygaard HB, Gilbert JW, Strittmatter SM. Cellular prion protein mediates impairment of synaptic plasticity by amyloid-β oligomers. Nature. fevereiro de 2009;457(7233):1128–32. doi:10.1038/nature07761

2. Chen S, Yadav SP, Surewicz WK. Interaction between Human Prion Protein and Amyloid-β (Aβ) Oligomers: ROLE OF N-TERMINAL RESIDUES *. J Biol Chem. 20 de agosto de 2010;285(34):26377–83. doi:10.1074/jbc.M110.145516 PubMed PMID: 20576610.

3. Fluharty BR, Biasini E, Stravalaci M, Sclip A, Diomede L, Balducci C, et al. An N-terminal Fragment of the Prion Protein Binds to Amyloid-β Oligomers and Inhibits Their Neurotoxicity in Vivo*. J Biol Chem. 15 de março de 2013;288(11):7857–66. doi:10.1074/jbc.M112.423954

4. Amin L, Harris DA. Aβ receptors specifically recognize molecular features displayed by fibril ends and neurotoxic oligomers. Nat Commun. 8 de junho de 2021;12(1):3451. doi:10.1038/s41467-021-23507-z PubMed PMID: 34103486; PubMed Central PMCID: PMC8187732.

5. Taylor DR, Hooper NM. The prion protein and lipid rafts (Review). Mol Membr Biol. 1º de janeiro de 2006;23(1):89–99. doi:10.1080/09687860500449994

6. Um JW, Nygaard HB, Heiss JK, Kostylev MA, Stagi M, Vortmeyer A, et al. Alzheimer amyloid-β oligomer bound to postsynaptic prion protein activates Fyn to impair neurons. Nat Neurosci. setembro de 2012;15(9):1227–35. doi:10.1038/nn.3178

7. Haas LT, Salazar SV, Smith LM, Zhao HR, Cox TO, Herber CS, et al. Silent Allosteric Modulation of mGluR5 Maintains Glutamate Signaling while Rescuing Alzheimer's Mouse Phenotypes. Cell Rep. 5 de julho de 2017;20(1):76–88. doi:10.1016/j.celrep.2017.06.023 PubMed PMID: 28683325.

8. Niswender CM, Conn PJ. Metabotropic Glutamate Receptors: Physiology, Pharmacology, and Disease. Annu Rev Pharmacol Toxicol. 10 de fevereiro de 2010;50(Volume 50, 2010):295–322. doi:10.1146/annurev.pharmtox.011008.145533

9. Resh MD. Myristylation and palmitylation of Src family members: The fats of the matter. Cell. 11 de fevereiro de 1994;76(3):411–3. doi:10.1016/0092-8674(94)90104-X PubMed PMID: 8313462.

10. Sato I, Obata Y, Kasahara K, Nakayama Y, Fukumoto Y, Yamasaki T, et al. Differential trafficking of Src, Lyn, Yes and Fyn is specified by the state of palmitoylation in the SH4 domain. J Cell Sci. 1º de abril de 2009;122(7):965–75. doi:10.1242/jcs.034843

11. Um JW, Nygaard HB, Heiss JK, Kostylev MA, Stagi M, Vortmeyer A, et al. Alzheimer amyloid-β oligomer bound to postsynaptic prion protein activates Fyn to impair neurons. Nat Neurosci. setembro de 2012;15(9):1227–35. doi:10.1038/nn.3178

12. Guglietti B, Sivasankar S, Mustafa S, Corrigan F, Collins-Praino LE. Fyn Kinase Activity and Its Role in Neurodegenerative Disease Pathology: a Potential Universal Target? Mol Neurobiol. 1º de novembro de 2021;58(11):5986–6005. doi:10.1007/s12035-021-02518-3

13. Tomasi V. Signal transduction in neurons: effects of cellular prion protein on fyn kinase and ERK1/2 kinase. Immun Ageing. 16 de dezembro de 2010;7(1):S5. doi:10.1186/1742-4933-7-S1-S5

14. Lee G, Newman ST, Gard DL, Band H, Panchamoorthy G. Tau interacts with src-family non-receptor tyrosine kinases. J Cell Sci. 1º de novembro de 1998;111(21):3167–77. doi:10.1242/jcs.111.21.3167

15. Ittner LM, Ke YD, Delerue F, Bi M, Gladbach A, Eersel J van, et al. Dendritic Function of Tau Mediates Amyloid-β Toxicity in Alzheimer's Disease Mouse Models. Cell. 6 de agosto de 2010;142(3):387–97. doi:10.1016/j.cell.2010.06.036 PubMed PMID: 20655099.

16. Thomas SM, Brugge JS. CELLULAR FUNCTIONS REGULATED BY SRC FAMILY KINASES. Annu Rev Cell Dev Biol. 1º de novembro de 1997;13(Volume 13, 1997):513–609. doi:10.1146/annurev.cellbio.13.1.513

17. Tezuka T, Umemori H, Akiyama T, Nakanishi S, Yamamoto T. PSD-95 promotes Fyn-mediated tyrosine phosphorylation of the N-methyl-D-aspartate receptor subunit NR2A. Proc Natl Acad Sci U S A. 19 de janeiro de 1999;96(2):435–40. doi:10.1073/pnas.96.2.435 PubMed PMID: 9892651; PubMed Central PMCID: PMC15154.

18. Ingley E. Src family kinases: Regulation of their activities, levels and identification of new pathways. Biochim Biophys Acta BBA - Proteins Proteomics. 1º de janeiro de 2008;Inhibitors of Protein Kinases (5th International Conference, IPK-2007) and Workshop Session on Molecular Design and Simulation Methods (Warsaw, Poland, June 23-27, 2007)1784(1):56–65. doi:10.1016/j.bbapap.2007.08.012

19. Bhandari V, Lim KL, Pallen CJ. Physical and Functional Interactions between Receptor-like Protein-tyrosine Phosphatase α and p59fyn*. J Biol Chem. 10 de abril de 1998;273(15):8691–8. doi:10.1074/jbc.273.15.8691 PubMed PMID: 9535845.

20. Harder KW, Moller NPH, Peacock JW, Jirik FR. Protein-tyrosine Phosphatase α Regulates Src Family Kinases and Alters Cell-Substratum Adhesion *. J Biol Chem. 27 de novembro de 1998;273(48):31890–900. doi:10.1074/jbc.273.48.31890 PubMed PMID: 9822658.

21. Lee G, Newman ST, Gard DL, Band H, Panchamoorthy G. Tau interacts with src-family non-receptor tyrosine kinases. J Cell Sci. 1º de novembro de 1998;111(21):3167–77. doi:10.1242/jcs.111.21.3167

22. Nakazawa T, Komai S, Tezuka T, Hisatsune C, Umemori H, Semba K, et al. Characterization of Fyn-mediated Tyrosine Phosphorylation Sites on GluRε2 (NR2B) Subunit of the N-Methyl-d-aspartate Receptor *. J Biol Chem. 5 de janeiro de 2001;276(1):693–9. doi:10.1074/jbc.M008085200

23. Prybylowski K, Chang K, Sans N, Kan L, Vicini S, Wenthold RJ. The Synaptic Localization of NR2B-Containing NMDA Receptors Is Controlled by Interactions with PDZ Proteins and AP-2. Neuron. 15 de setembro de 2005;47(6):845–57. doi:10.1016/j.neuron.2005.08.016 PubMed PMID: 16157279.

24. Hardingham GE, Bading H. Synaptic versus extrasynaptic NMDA receptor signalling: implications for neurodegenerative disorders. Nat Rev Neurosci. outubro de 2010;11(10):682–96. doi:10.1038/nrn2911

25. Hernandez P, Lee G, Sjoberg M, Maccioni RB. Tau Phosphorylation by cdk5 and Fyn in Response to Amyloid Peptide Aβ25–35: Involvement of Lipid Rafts. J Alzheimer's Dis. 4 de janeiro de 2009;16(1):149–56. doi:10.3233/JAD-2009-0933

26. Sontag JM, Sontag E. Protein phosphatase 2A dysfunction in Alzheimer's disease. Front Mol Neurosci. 11 de março de 2014;7. doi:10.3389/fnmol.2014.00016

27. Cross DAE, Alessi DR, Cohen P, Andjelkovich M, Hemmings BA. Inhibition of glycogen synthase kinase-3 by insulin mediated by protein kinase B. Nature. dezembro de 1995;378(6559):785–9. doi:10.1038/378785a0

28. Sutherland C. What Are the bona fide GSK3 Substrates? Int J Alzheimer's Dis. 2011;2011(1):505607. doi:10.4061/2011/505607

29. Patrick GN, Zukerberg L, Nikolic M, de la Monte S, Dikkes P, Tsai LH. Conversion of p35 to p25 deregulates Cdk5 activity and promotes neurodegeneration. Nature. dezembro de 1999;402(6762):615–22. doi:10.1038/45159

30. AVILA J, LUCAS JJ, PÉREZ M, HERNÁNDEZ F. Role of Tau Protein in Both Physiological and Pathological Conditions. Physiol Rev. abril de 2004;84(2):361–84. doi:10.1152/physrev.00024.2003

31. Wang Y, Mandelkow E. Tau in physiology and pathology. Nat Rev Neurosci. janeiro de 2016;17(1):22–35. doi:10.1038/nrn.2015.1

32. Buée L, Bussière T, Buée-Scherrer V, Delacourte A, Hof PR. Tau protein isoforms, phosphorylation and role in neurodegenerative disorders1. Brain Res Rev. 1º de agosto de 2000;33(1):95–130. doi:10.1016/S0165-0173(00)00019-9

33. Lee G, Thangavel R, Sharma VM, Litersky JM, Bhaskar K, Fang SM, et al. Phosphorylation of Tau by Fyn: Implications for Alzheimer's Disease. J Neurosci. 3 de março de 2004;24(9):2304–12. doi:10.1523/JNEUROSCI.4162-03.2004 PubMed PMID: 14999081.

34. Iqbal K, del C. Alonso A, Chen S, Chohan MO, El-Akkad E, Gong CX, et al. Tau pathology in Alzheimer disease and other tauopathies. Biochim Biophys Acta BBA - Mol Basis Dis. 3 de janeiro de 2005;The Biology and Pathobiology of Tau1739(2):198–210. doi:10.1016/j.bbadis.2004.09.008

35. Zempel H, Luedtke J, Kumar Y, Biernat J, Dawson H, Mandelkow E, et al. Amyloid-β oligomers induce synaptic damage via Tau-dependent microtubule severing by TTLL6 and spastin. EMBO J. 1º de novembro de 2013;32(22):2920–37. doi:10.1038/emboj.2013.207

36. Balducci C, Orsini F, Cerovic M, Beeg M, Rocutto B, Dacomo L, et al. Tau oligomers impair memory and synaptic plasticity through the cellular prion protein. Acta Neuropathol Commun. 27 de janeiro de 2025;13(1):17. doi:10.1186/s40478-025-01930-3
